# Molecular Modeling of Myrosinase from *Brassica oleracea*: A Structural Investigation of Sinigrin Interaction

**DOI:** 10.3390/genes6041315

**Published:** 2015-12-21

**Authors:** Sathishkumar Natarajan, Senthil Kumar Thamilarasan, Jong-In Park, Mi-Young Chung, Ill-Sup Nou

**Affiliations:** 1 Department of Horticulture, Sunchon National University, Suncheon 57922, Korea; E-Mails: sathish@sunchon.ac.kr (S.N.); senkuttybio@gmail.com (S.K.T.); jipark@sunchon.ac.kr (J.-I.P.); 2 Department of Agricultural Education, Sunchon National University, Suncheon 540-950, Korea; E-Mail: queen@sunchon.ac.kr

**Keywords:** myrosinase, sinigrin, *Brassica oleracea*, homology modeling, docking and dynamics

## Abstract

Myrosinase, which is present in cruciferous plant species, plays an important role in the hydrolysis of glycosides such as glucosinolates and is involved in plant defense. *Brassicaceae* myrosinases are diverse although they share common ancestry, and structural knowledge about myrosinases from cabbage (*Brassica oleracea*) was needed. To address this, we constructed a three-dimensional model structure of myrosinase based on *Sinapis alba* structures using Iterative Threading ASSEmbly Refinement server (I-TASSER) webserver, and refined model coordinates were evaluated with ProQ and Verify3D. The resulting model was predicted with β/α *fold, ten conserved* N-glycosylation sites, and three disulfide bridges. In addition, this model shared features with the known *Sinapis alba* myrosinase structure. To obtain a better understanding of myrosinase–sinigrin interaction, the refined model was docked using Autodock Vina with crucial key amino acids. The key nucleophile residues GLN207 and GLU427 were found to interact with sinigrin to form a hydrogen bond. Further, 20-ns molecular dynamics simulation was performed to examine myrosinase–sinigrin complex stability, revealing that residue GLU207 maintained its hydrogen bond stability throughout the entire simulation and structural orientation was similar to that of the docked state. This conceptual model should be useful for understanding the structural features of myrosinase and their binding orientation with sinigrin.

## 1. Introduction

Myrosinase (also termed thioglucoside glucohydrolase, sinigrinase, or sinigrase EC: 3.2.1.147), is an S-glycosidase involved in breakdown of thioglucosides such as glucosinolates (GSL), a large group of secondary metabolites especially abundant in cruciferous plants. Myrosinase is localized in specialized GSL-free idioblast cells termed myrosin cells [1]. GSLs, classified based on their structure as indolic or aliphatic, are biologically inactive in their native state; myrosinase catalyzes the hydrolysis of these thioglucosides into d-glucose and a thiol (sulfate) such as aglycone (thiohydroximate-*O*-sulfonate). The thiols produced are unstable intermediates that differ mainly based on side chain structures, myrosinase substrate, plant species, and other reaction conditions like pH or availability of ferrous ions and environmental factors [2,3,4]. Compounds spontaneously formed from aglycone in this process are biologically intact, active and sometimes toxic, and include isothiocyanates and nitrile, epithionitriles, thiocyanates, and oxazoldine-thione [5], which are involved in plant defense against herbivory, pathogen invasion and tissue damage [1,3]. Concentrations of GSLs vary among different parts of the plant [6,7]. External factors such as tissue damage and pathogen attack trigger the release of GSLs, which results in interaction with myrosinase and eventually degradation of the glycosides. In plants, myrosinase activities vary between species, cultivars, and parts of the plant examined. Myrosinase activity is highest in seeds and seedling stages as indolic GSLs are broken down to form indoleacetic acid (IAA), a phytohormone essential for developing plants [8]. In cruciferous species, the interaction between myrosinase and GSLs for defense creates a so-called binary mustard oil bomb. These myrosinase are activated by ascorbic acid and the three-dimensional structures of several binary and ternary complexes of the enzyme with different transition state analogues, with ascorbic acid and with inhibitors mimicking the substrate have been reported. In addition, mechanistic details already known include the position of a water molecule placed perfectly for activation by ascorbate and for nucleophilic attack on the covalent intermediate of the reaction [9]. Biochemical, genetic and phylogenetic data, [10] reported that the myrosinase-GSL system evolved from cyanogenic glucosides and *O*-β-glucosidases. Products of GSLs possess toxic and pharmacological effects on a wide range of organisms [11,12]. GSLs induce Phase I and Phase II detoxifying enzymes during cancer infection and alter steroid hormone levels during their metabolism, in turn aiding in protecting the cell from oxidative damage [13]. Cruciferous species are known for the presence of GSLs, conferring their chemo-preventive effects [14]. Broccoli (*Brassica oleracea var. italica*) was reported to act against and provide protection from liver cancer in animals [15]. In humans, glycosides are involved in reducing DNA damage in lymphocytes [16,17]. There was significant anti-nutritional activity in animals when they were fed with GSLs [14]. GSLs from plants have become an interesting area of work due to their benefits to animal and human health such as anti-microbial [18], anti-nematode [19] insecticidal, weed-inhibiting [20], herbicidal [10,21], allelopathic [22], and anticarcinogenic [15,23] activities. To date, more than 130 GSLs have been identified and reported in plants, of which more than 30 are found in the *Brassicaceae* family [24]. Lenman *et al* [25] reported that myrosinase shows differential expression throughout development and also in various parts of the plant. Molecular breeders have used traditional breeding methods to increase the content of GSLs due to their beneficial activities. This study aims to improve understanding of myrosinase–sinigrin interaction at the molecular level. To date, one conceptual model was constructed from *Brassica juncia* and described their myrosinase structural features [26]. Homology modeling is a powerful method to predict three-dimensional structures from amino acid sequences [27]. This approach can be used to obtain structural knowledge of proteins, enzyme-substrate interactions, and protein-ligand interactions [28,29]. In this study, we have applied this technique to predict the structure of myrosinase from *Brassica oleracea* (*B. oleracea*, wild cabbage) and examine its interaction with sinigrin, one of the possible substrates.

## 2. Materials and Methods

### 2.1. Template Identification and Homology Modeling

The amino acid sequence of myrosinase (MYR) from cabbage (*B. oleracea*, wild cabbage) was obtained from the NCBI protein sequence database (Accession number: ABS30827) [30]. The primary sequence of MYR protein from cabbage (*B. oleracea*) consists of 546 amino acids, which was used for modeling. Homology modeling was performed using the Iterative Threading ASSEmbly Refinement (I-TASSER) server based on the *ab initio*/threading method [31]. To predict the three-dimensional structure of cabbage (*B. oleracea*) MYR, 1MYR_A (*Sinapis alba* (*S. alba*) MYR) [32], 1E4M_M (*S. alba* MYR) [9], 1DWF_M (*S. alba* MYR) [33], 1VO2_A (*Sorghum bicolor* Dhurrinase) [34], and 4JHO_A (*Oryza sativa* beta-glucosidase) [35] structures were chosen as the templates. These template structures were selected by multiple threading approach Local Meta-Threading-Server (LOMETS) based on sequence similarity to cabbage (*B. oleracea*) MYR. Alignments of the query sequence with each template were generated using the ConSurf server. This program provides multiple sequence alignment with conserved features, which provides information about specific amino acid features in their local environment [36]. A total of five three-dimensional (3D) models were generated by I-TASSER; among them, the best model was identified based on confidence score (C-Score) [37].

### 2.2. Molecular Dynamics Simulation

Simulations of molecular dynamics (MD) were performed for the modeled MYR protein using the GROMOS96 43a1 force field implemented in GROningen MAchine for Chemical Simulation (GROMACS) version 4.6 [38,39]. A cubic box was constructed around the 3D model with a minimum of 10 Å between the box edges and the model surface. Appropriate counter-ions (Na^+^ or Cl^−^) were added for neutralizing this system. Further it was solvated with simple point charge (SPC) water molecules and minimized using the steepest descent method to remove spurious contacts. In addition, this system was equilibrated 100 ps under NVT (canonical ensemble) and NPT (isothermal-isobaric ensemble) conditions. The temperature of the system was set to 300 K after applying position restrains to the protein. This temperature and pressure was maintained using the Berendsen weak-coupling method. A cut-off radius of 0.9 nm was used in the simulation for van der Waals and electrostatic interactions [40]. The LINCS algorithm was used to reduce close contact between the complex system and bond constraints [41]. Finally, a 20-ns production was carried out for the MYR model using the particle mesh Ewald (PME) electrostatics method under NPT conditions. For analysis, final coordinates were saved every 2 ps. The final MYR trajectory coordinates of the 20-ns simulation were selected, subjected to energy minimization. Further, the refined model was validated using Protein Quality Predictor (ProQ) [42] and VERIFY3D plot [43] and then used for molecular docking studies.

### 2.3. Analysis of MYR Active Site and N-Glycosylation Sites

The refined model was analyzed using Catalytic Site Atlas (CSA) database [16] and Q-SiteFinder [44] for active site identification. Those sites were compared with template structures for structural evaluation. Further *N*-glycosylation sites were identified using the NetNGlyc1.0 server [45].

### 2.4. MYR–Sinigrin Interaction

The two-dimensional (2D) structure of sinigrin (2-propenylglucosinlate) was retrieved from the NCBI PubChem database with the following accession number CID: 5464493. This molecule was drawn using ChemSketch (Advanced Chemistry Development, Inc. Toronto, ON, Canada), saved in a 2D (.mol) format, and then converted into a 3D (.pdb) format by importing into Discovery Studio 3.5 visualizer (DS 3.5) (DS, Accelrys, Inc. SanDiego, CA, USA). This molecule was optimized using the Conjugate Gradients method [46] followed by Steepest Descent in 200 steps using the PyRx program [47]. Universal Force Field (UFF) was used for minimization of the sinigrin molecule [48]. Molecular docking analysis was performed using AutoDock Vina [49]. Active site residues were carefully assigned to the binding pocket of the MYR protein, and docking steps were followed from previous studies [50]. The best docking orientation was identified from binding affinity score and hydrogen bond interaction to the active site based on visual inspection. The 2D graphical visualization of MYR and sinigrin interaction was generated by DS 3.5. The X-Score program [51] was used to calculate binding affinity scores of sinigrin and non-bonded interactions.

### 2.5. Complex Stability Refinement by Molecular Dynamics Simulation

The MYR–sinigrin docked-complex file obtained from docking studies was subjected to MD using GROMACS 4.6. The sinigrin structural topology file was generated using the Dundee PRODRG2 server [52] with chirality, full charges and energy minimization. In addition, MYR topology was created using the GROMACS pdb2gmx utility. The above-mentioned dynamics parameter was used to produce a 20-ns simulation for the modeled MYR and MYR–sinigrin complex. The detailed MD procedure was implemented according to our previous study [51]. In this analysis, root mean square deviation (RMSD), and root mean square fluctuation (RMSF) calculations were computed using the GROMACS utilities of g_rms, and g_rmsf, respectively. For this study, Chimera [53], and DS 3.5 were used for graphical visualization, and graphs were plotted using Microsoft Excel.

## 3. Results and Discussion

### 3.1. Homology Modeling

**Figure 1:**
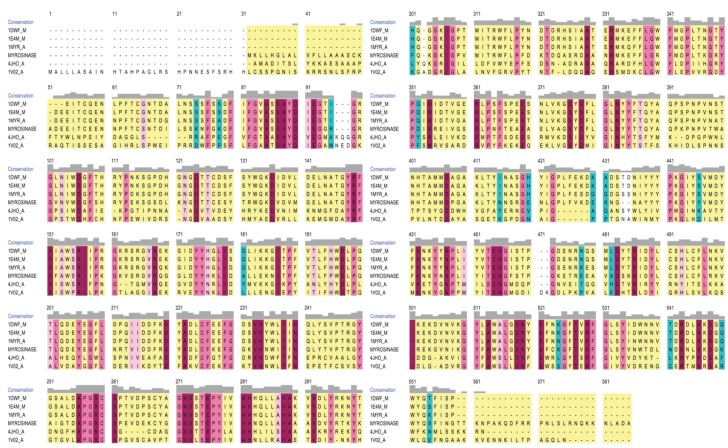
Sequence alignment of myrosinase with their template structures generated by using Chimera visualization tool. Gaps and conservation residues were denoted in dotted lines and gray color, respectively. In addition, block levels represented as identical (tallest blocks), conserved (Intermediate blocks) and mild conserved (Small blocks) and not identical (no blocks).

The full-length cabbage (*B. oleracea*) MYR protein consists of 546 amino acids including a glycoside hydrolase family 1 (Glyco_hydro_1) domain (41–522 amino acids). A search for homologous proteins for modeling cabbage (*B. oleracea*) MYR prompted us to use 1MYR_A, 1E4M_M, 1DWF_M, 1VO2_A and 4JHO_A protein structures as templates. Multiple sequence alignment was performed with these template proteins to identify sequence alignments of structural motifs (Figure 1). The alignment results revealed good conservation of cabbage (*B. oleracea*) MYR with the *S. alba* proteins, with 91% query coverage and 72% sequence identity. Cabbage (*B. oleracea*) MYR shared 40% identity with the protein from *Sorghum bicolor* (1VO2_A) and 39% identity with that of *Oryza sativa* (4JHO_A). In addition, three potential *S. alba* templates were found to share their common ancestor with cabbage (*B. oleracea*) MYR. The best model was selected based on C-Score value, and this score was used for estimating the quality of predicted models by I-TASSER. Then, the selected best model was subjected to MD Simulations.

### 3.2. Model Refinement and Structure of MYR

**Figure 2:**
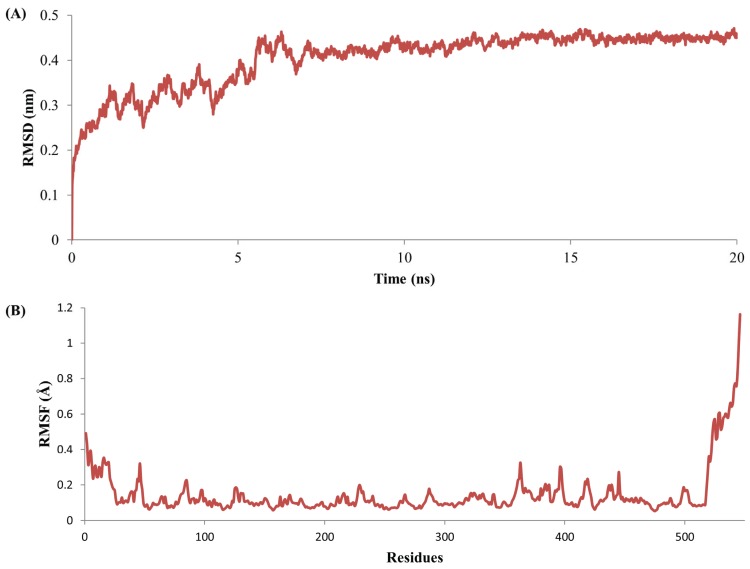
Molecular dynamics based myrosinase model refinement through 20 ns simulation: (**A**) The root mean square deviation (RMSD) values of backbone and (**B**) root mean square fluctuations (RMSF) values of atoms.

**Figure 3:**
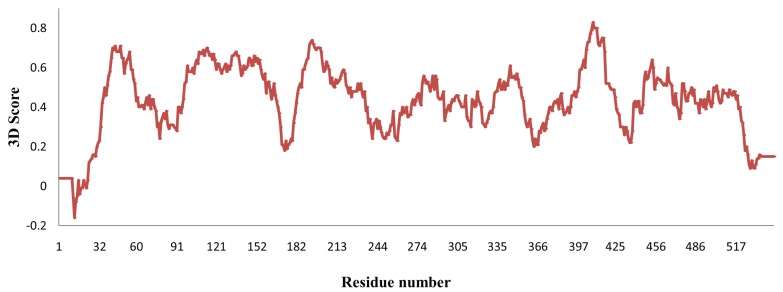
The 3D scores for the modeled myrosinase structure from *Brassica oleracea*.

The stability and MD properties of the constructed model were evaluated using MD simulations. To identify the energetically favorable structure for this model, MD simulation was observed up to 20 ns. The RMSD values were examined throughout the entire simulation time to check the structural stability of the tested model. RMSD values of MYR backbone atoms attained equilibration state in approximately 7.1 ns with an average of 0.43Å and remained stable throughout the course of the simulation (Figure 2A). RMSF analyses of backbone atoms of each amino acid are shown in Figure 2B; much structural variation were found in secondary structure. Numerous reports have confirmed that O-glycosidase family enzymes are linked to a single (β/α) _8_ barrel [54]. A final snapshot from the 20-ns MD simulation was subjected to further evaluation and refinement. The stereo-chemical quality of the enhanced model was validated by Veryfy3D and ProQ. The Verify3D evaluation server reported that our optimized model scored over 80% of amino acids in the 3D/1D profile, which indicates residues are in favorable positions (Figure 3). ProQ revealed an LGscore of 4.808, which indicates that our conceptual MYR structure was an extremely good model. From these evaluation results, we confirmed that the developed model was satisfactory, and we therefore used it for subsequent analyses.

Furthermore, this refined MYR protein was compared with template structures to identify sequence conservation. The alignment results revealed that our modeled Cabbage (*B. oleracea*) MYR shared with sequence conservation of 72.14%, 71.54%, 71.54%, 40.29%, and 39.13% with 1MYR_A, 1DWF_M, 1E4M_M, 1VO2_A, and 4JHO_A structures, respectively. In addition, the three templates from *S.alba* (1MYR_A, 1DWF_M, and 1E4M_M) were shown high similarity with refined MYR including Glyco_hydro_1 domain. The GOR4 analysis has revealed that our cabbage (*B. oleracea*) MYR model contains secondary structural elements of 22.71% helix, 20.51% strand, and 56.78% coil. Three disulfide bridges were found, in the Cys2–Cys456, Cys34–Cys454, and Cys226–Cys234 regions. The known myrosinase crystal structure from *S. alba* has shown similar structural features [26]. Diverse myrosinase proteins are involved in plant defense mechanisms, and although their origins vary, they are associated with common ancestors. Moreover, *N*-glycosylation sites are important to maintain the molecular stability and solubility of myrosinase in the dehydrated environment. *N*-glycosylation sites were predicted for the optimized MYR model using the NetNGlyc1.0 server. A total of ten Asn-Xaa-Ser/Thr sites were observed, at residues 110, 152, 177, 238, 329, 366, 380, 497, 518, and 533. Among these potential *N*-glycosylation sites, those with a threshold value over 0.5 were considered as potential sites and the residues were 110, 177, 329, 497, and 533. In comparison, ten sites were predicted in *S. alba* (six potential) and seven were reported from a theoretical model of *Brassica juncea* (*B. juncea*) [26]. The number of predicted *N*-glycosylation sites from cabbage *(B. oleracea*) was close to *S. alba*, in accordance with good amino acid conservations including potential sites (Supplementary Figure S1).

### 3.3. Molecular Interaction Studies

**Figure 4:**
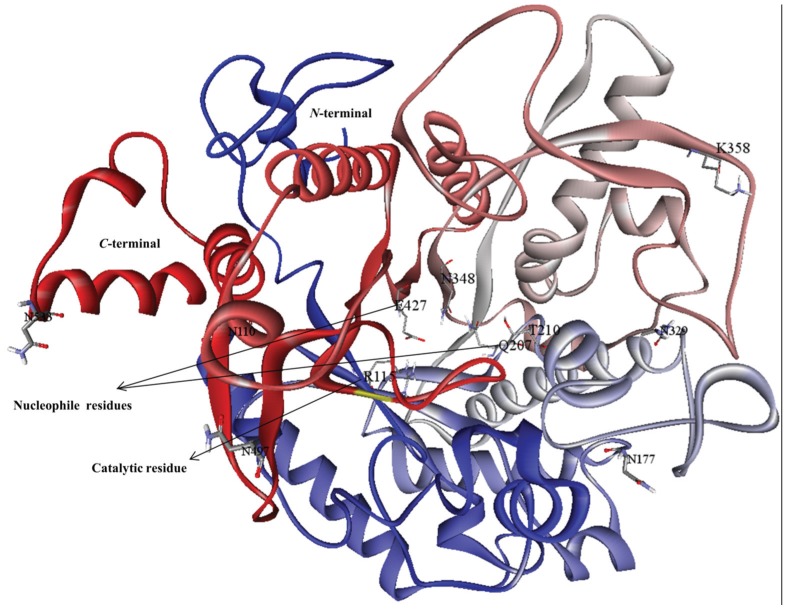
Three-dimensional structure of modeled myrosinase from *Brassica oleracea*. This figure was generated using DS 3.5 visualizer and colors of N-to-C terminal are based on their secondary structure.

**Figure 5:**
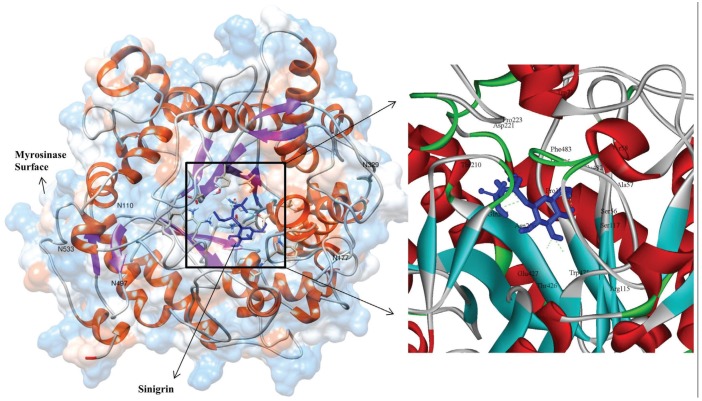
Graphical illustration of docked complex of myrosinase protein with sinigrin and potential *N*-glycosylation sites were highlighted. The 2D plot of interaction results were denoted as in black color (interacted amino acids), dotted green lines (hydrogen bonds) and blue color (sinigrin).

To better understand MYR from cabbage (*B. oleracea*), an interaction study between the substrate sinigrin and the enhanced MYR protein active sites was carried out using the Autodock Vina program. For docking studies, potential active site information was examined from the *S. alba* myrosinase crystal structure and confirmed with the CSA database and Q-site finder. Residue ARG115 was involved in the catalytic target site, and GLN207 and GLU427 had catalytic nucleophile activities. In addition, THR210, ASN348, and TYR350 were present in the binding pocket of MYR for substrate binding (Figure 4). These key amino acids were assigned as active sites in docking to identify productive binding orientations. Three-dimensional docked representations of the sinigrin molecule in the MYR active site and interacted 2D plot are shown in Figure 5. Further, myrosinase–sinigrin interaction was evaluated by binding affinity scores and hydrogen bond formation in the MYR active sites. In the docked state, the substrate sinigrin formed hydrogen bonds with the catalytic target site and two catalytic nucleophile residues, along with other residues of SER117, ASP221, and LYS285 (Table 1). The docking program predicted that MYR–sinigrin binding affinity was −7.2 kcal/mol in the docked state. The nucleophile residue GLN207 was involved in the formation of one hydrogen bond to the HE21-O sinigrin group with hydrogen bond length of 2.10Å. A second nucleophilic residue, GLU427, formed two separate hydrogen bonds with the hydrogen atoms present in the sinigrin (H-OE1, and H-OE2) with hydrogen bond length of 1.85Å and 2.34Å respectively. In addition, one hydrogen bond was observed at the residue SER117 HG atom (HG-O) with 1.7Å hydrogen bond length. We also observed two hydrogen bonds with LYS485 (HZ2-O and HZ2-O) with lengths of 2.43Å and 2.41Å, respectively. Moreover, sinigrin contains seven rotatable bonds for enzyme binding, including one nitrogen (N) atom and sulfur group. This specific sulfur group was used to recognize the MYR binding and complex stability. In the docked state, residues GLU427 and LYS485 interacted with N atoms and the sulfur group respectively. In addition, the following hydrophobic interaction residues also contributed to molecular stability: TRP475, THR426, ASN206, PHE483, GLU482, SER56, ALA57, PRO161, TRP71, TYR58, and PRO223.

**Table 1:** Hydrogen bond interaction results for *B. oleracea* MYR–sinigrin.

S. No	Hydrogen Bond Interacting Residue	Hydrogen Bond Donor	Hydrogen Bond Acceptor	Hydrogen Bond Length (Å)	Number of Hydrogen Bonds
1	ARG115 *	ARG115:HH22	LIG1:O	2.36	1
2	SER117	SER117:HG	LIG1:O	1.7	1
3	GLN207 *	GLN207:HE21	LIG:O	2.10	1
4	T221	LIG1:HN	ASP221:OD1	2.27	1
5	GLU427 *	LIG1:H	GLU427:OE1	1.85	2
		LIG1:H	GLU427:OE2	2.34	-
6	LYS485	LYS485:HZ2	LIG1:O	2.43	2
		LYS485:HZ2	LIG1:O	2.41	-
Non-bonded interacted residues: TRP475, THR426, ASN206, PHE483, GLU482, SER56, ALA57, PRO161, TRP71, TYR58 AND PRO223					
* Catalytic and nucleophile residues are highlighted in bold.					

### 3.4. Stability Evolution of the MYR–Sinigrin Complex

**Figure 6:**
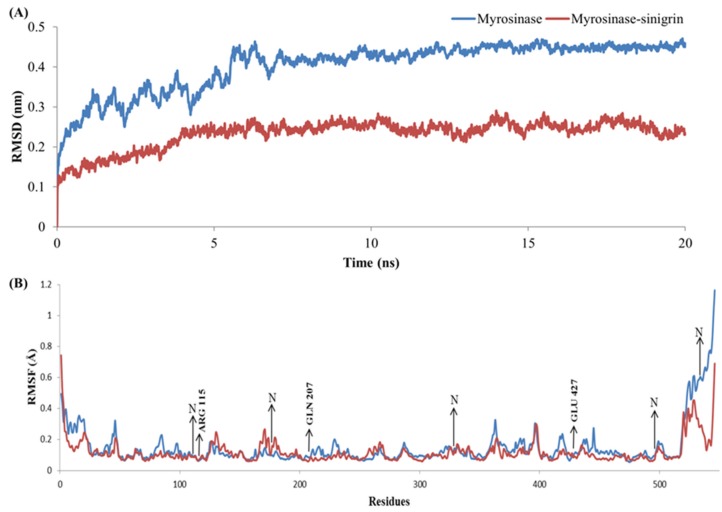
The root mean square deviation (RMSD) values of backbone (**A**) and root mean square Flucyuations (RMSF) values of atoms (**B**) for myrosinase and myrosinase-sinigrin complex during 20 ns dynamic simulations.

An MD simulation was conducted to examine the MYR–sinigrin complex stability, and differences were monitored by RMSD with (holo) and without (apo) sinigrin. Accordingly, two individual MD simulations were carried out; the first contained only MYR as discussed above, the second consisted of the MYR–sinigrin complex file that was obtained from docking analysis. The protein mobility and structural changes were measured by RMSD plot of MYR backbone atoms throughout the 20-ns simulation. RMSD analysis of the MD trajectory noted that fluctuations were attained to 6.20 ns with an average of 0.26 Å, and then retained stability throughout simulation (Figure 6A). Apo and holo forms of MYR attained equilibration at over 6 ns. RMSD values of the apo form ranged between 0.0005 and 0.4711 Å, which was higher than those of the holo form, which ranged between 0.0005 Å and 0.2918 Å during 20 ns trajectories. Further, RMSF values were calculated for the apo and holo forms of MYR to estimate local protein mobility. Analysis of both RMSF values against backbone atoms indicated that a few atoms highly fluctuated in the *N*-terminal and *C*-terminal ends. In addition, the backbone atoms of RMSF values were compared with MYR (apo) and MYR-sinigrin complex (holo) (Figure 6B). Analyzing the trajectory files, the crucial residues had RMSF values ranging from 0 to 0.11 Å. In addition, compare to apo form of MYR, nucleophilic and catalytic residues namely GLN207, GLU427, and ARG115 were seen with slight fluctuations. The results from both apo and holo form of MYR showed that the residues in the sinigrin binding regions stabilized upon ligand recognition. Further, higher fluctuations in RMSF values were noted at *N*-terminal and *C*-terminal than other regions. The superimposition of initial and final structure revealed that sinigrin-binding orientation was well preserved and slight structural changes were observed in the active sites. Moreover the RMSF values of predicted ten *N*-glycosylation sites were ranged between 0.08 Å and 0.28 Å over 20-ns simulation time (Supplementary Figure S2). Analysis of potential *N*-glycosylation sites revealed that slight and higher fluctuations were observed at the residues of 110, 117, 329, and 497 and 533, respectively, when compared to apo and holo form of MYR. The final trajectory of the 20-ns simulation was examined to predict binding affinity and hydrogen bond stability. Hydrogen bond stability analysis showed that the nucleophile GLN207 maintained the hydrogen bond throughout the entire 20-ns simulation (Supplementary Figure S3). This nucleophilic residue is responsible for enzyme-substrate interaction [55,56]. Another active site residue, THR210, formed a hydrogen bond in the hydrophobic state. By contrast, the hydrogen bonds of residues SER117, THR221, and LYS485 disappeared compared to the docking state in long dynamic simulations. We calculated the binding affinity of MYR-sinigrin to be −6.98 kcal/mol using the X-Score program (Table 2). In the case of non-bonded interaction, we observed negative logarithm dissociation constants of hydrophobic pair score (5.12), hydrophobic match score (5.13), hydrophobic surface score (5.11) and predicted mean binding affinity score (5.12). Both docking and MD simulation results suggests that the substrate sinigrin successfully recognized the MYR binding pocket at crucial active sites.

In general, conceptual model has been developed based on homology principle and help to characterize their putative functions in preliminary way. In addition, this structure based homology prediction method has limitations at the structural level, including large protein, multi-complex, and multi-domain proteins [57]. The developed model will be helpful to characterize the myrosinase function and sinigrin interaction at molecular level but further experimental confirmations are required.

**Table 2:** The bonded and non-bonded interaction scores of MYR–sinigrin complex after 20-ns molecular dynamics simulations. Scores were calculated using the X-score program.

Molecule	Predicted Binding Affinity (kcal/mol)	Hydrophobic Pair Score (p*K*d)	Hydrophobic Match Score (p*K*d)	Hydrophobic Surface Score (p*K*d)	Predicted Mean Binding Affinity (p*K*d)
MYR–sinigrin complex	−6.98	5.12	5.13	5.11	5.12

## 4. Conclusions

In this study, we constructed 3D model of MYR from cabbage (*B. oleracea*) based on sequence and structural similarity. The constructed model, which includes the Glyco_hydro_1 domain, was used for MD simulation to evaluate protein structural stability. The stereo-chemical quality of the optimized model confirmed that all residues were in favorable positions (Veryfy3D) and that the model was extremely good (ProQ). Our refined model included three disulfide bridges at the following positions: Cys2–Cys456, Cys34–Cys454, and Cys226–Cys234. In addition, ten conserved Asn-Xaa-Ser/Thr *N*-glycosylation sites were detected, among them five residues were potential sites, having a threshold value over 0.5. Notably, *N*-glycosylation sites, active sites and sequence conservation of our conceptual model showed similarity to the *S. alba* MYR crystal structure. Further, molecular interaction analysis showed two nucleophile (GLN207, and GLU427) and one catalytic (ARG115) residues were involved in the formation of hydrogen bonds along with hydrophobic interactions. The specific sulfur and nitrogen atoms present in sinigrin were well recognized by MYR active sites in the docked state. MD simulation results also suggested that strong hydrogen bond formation was maintained at residue GLU207 throughout the entire simulation, and binding orientation are the same as observed in the initial MYR–sinigrin complex structure. Our proposed 3D model of MYR and sinigrin binding orientation will be helpful to understand the MYR structural activities.

## Supplementary Files

Supplementary File 1
