# Supplementary material for: Molecular Modeling of Myrosinase from Brassica oleracea: A Structural Investigation of Sinigrin Interaction
Source: Genes (Basel). 2015 Dec 21;6(4):1315–29. doi: 10.3390/genes6041315 (PMC4690043; doi:10.3390/genes6041315)
Supplement: Supplementary File 1 [file genes-06-01315-s001.pdf]

Supplementary Materials

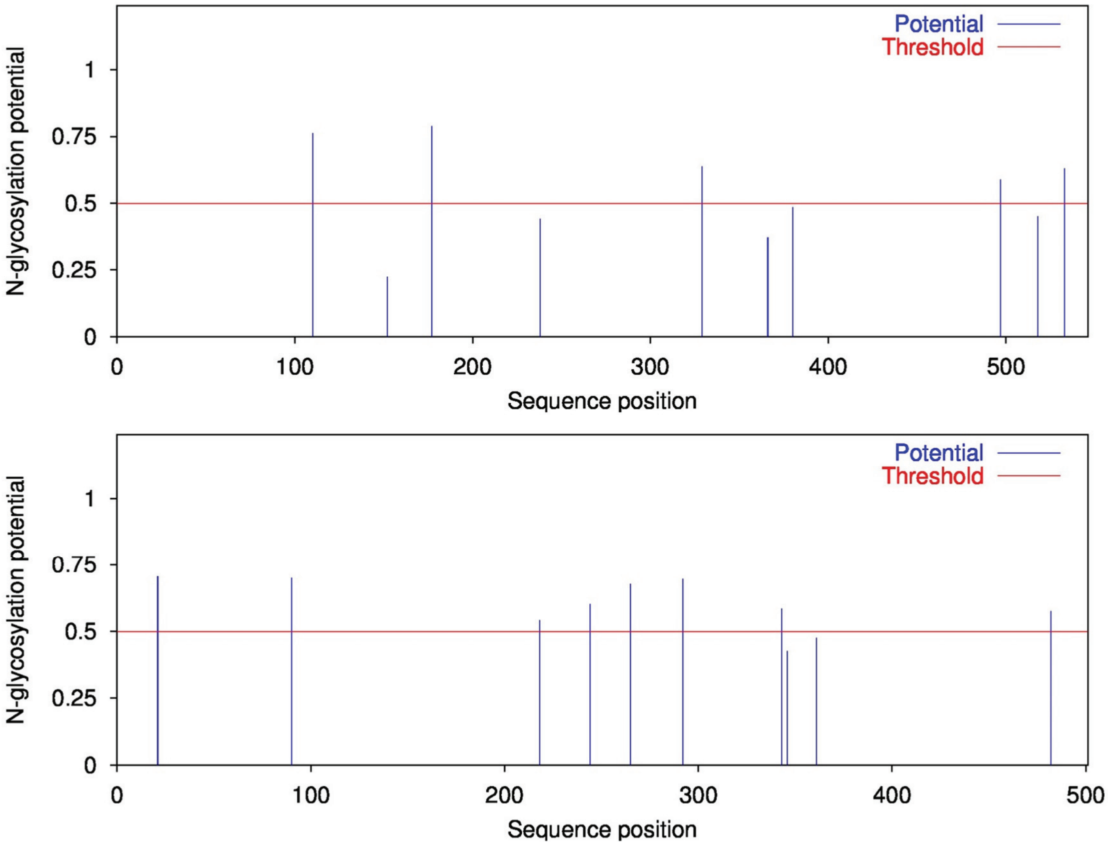

Figure S1. *N*-glycosylation plots of modeled myrosinase from *B. oleracea* (**A**) and myrosinase crystal structure from *S. alba* (**B**). Predicted sites were denoted in blue lines along with sequence position and over 0.5 threshold value considered as potential sites.

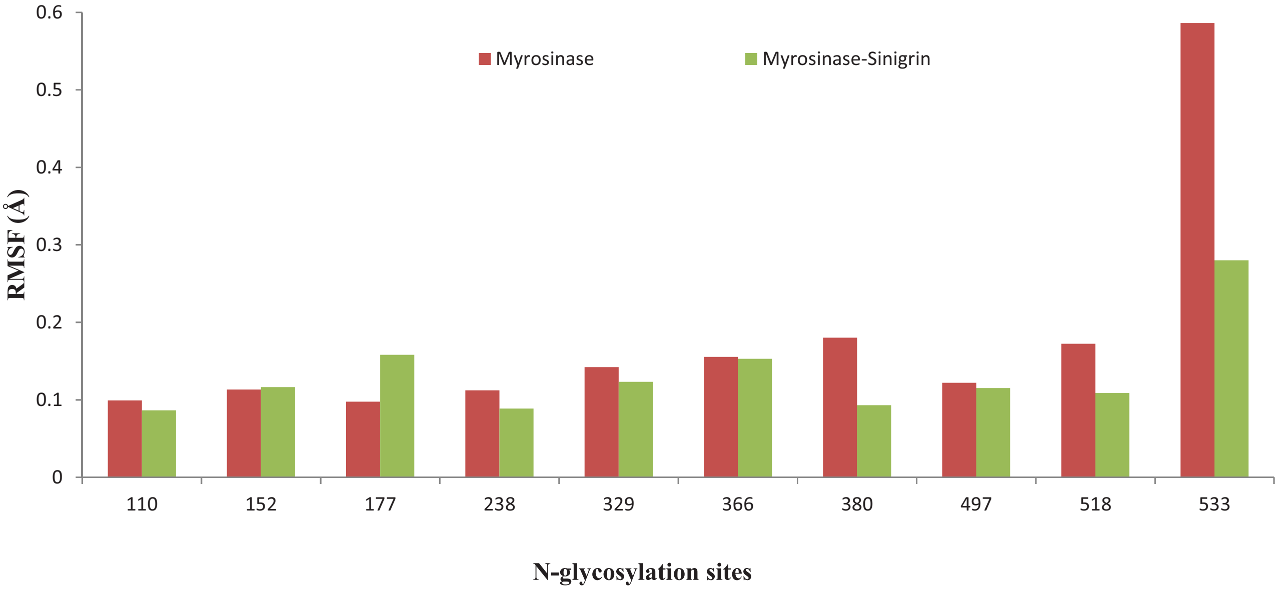

Figure S2. RMSF plot of ten predicted *N*-glycosylation sites obtained over 20 ns simulations. Potential *N*-glycosylation sites were denoted in asterisk (*) according to NetNGlyc1.0 server.

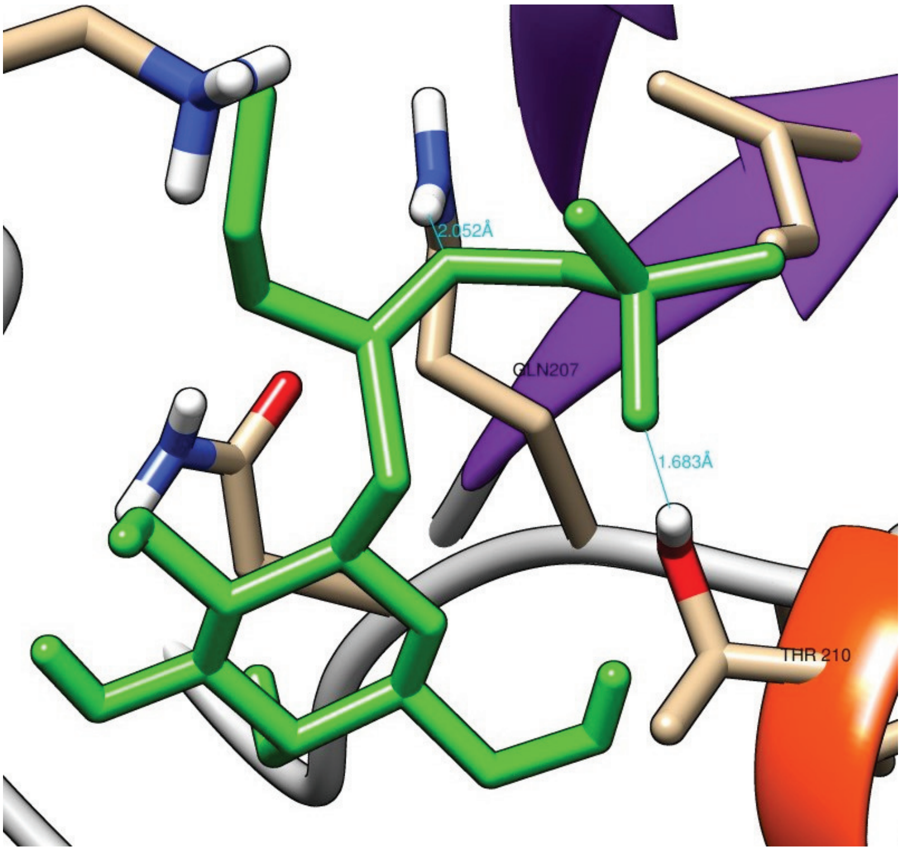

Figure S3. Illustration of the sinigrin interaction with the modeled 3D structure of myrosinase after 20 ns of MD simulation. The myrosinase were shown in ribbon form with their secondary structure color by UCSF chimera. The sinigrin were colored by green and their interacted residues and hydrogen bond distances were highlighted.

© 2015 by the authors; licensee MDPI, Basel, Switzerland. This article is an open access article distributed under the terms and conditions of the Creative Commons Attribution license (http://creativecommons.org/licenses/by/4.0/).
